# Adenoid cystic carcinoma in a male breast: a case report and literature review

**DOI:** 10.1093/jscr/rjaf226

**Published:** 2025-04-19

**Authors:** Vatsala Kapoor, Cara Moses, Sarah P Hanna, John W Odom

**Affiliations:** Medical College of Georgia, 1120 5th St, Augusta, GA 30912, United States; Department of Surgery, St. Josephs/Candler Health System, 11704 Mercy Boulevard, Savannah, GA 31419, United States; Medical College of Georgia, 1120 5th St, Augusta, GA 30912, United States; Department of Surgery, St. Josephs/Candler Health System, 11704 Mercy Boulevard, Savannah, GA 31419, United States

**Keywords:** male, breast, adenoid cystic carcinoma

## Abstract

Breast adenoid cystic carcinoma (BACC) is a rare occurrence constituting less than 0.1% of primary breast cancers. BACC is even more rare in male patients. Furthermore, there are currently no concrete treatment guidelines for BACC. Therefore, it is imperative to conduct a thoughtful evaluation and diagnostic workup when a potential case is encountered. We report a 63-year old male who initially presented to the dermatologist with a nonhealing inflamed leiomyoma of the left breast, raising suspicion for inflammatory breast cancer. A percutaneous biopsy was performed, and pathology revealed fragments of cribriform and papillary-like carcinoma, leading to a differential diagnosis including adenoid cystic carcinoma and basal-like ductal carcinoma *in situ*. A modified radical mastectomy was performed, and final surgical pathology revealed BACC. The patient did not receive any adjuvant therapy and is currently without any evidence of disease.

## Introduction

Breast adenoid cystic carcinoma (BACC) is an uncommon subtype of invasive breast carcinoma seen in less than 0.1% of primary breast cancers [1]. BACC in males is exceedingly rare, with nearly all available literature being case reports. Although BACC is typically negative for estrogen, progesterone, and human epidermal growth factor (HER2) receptors and triple-negative breast cancers normally hold a poor prognosis [2], BACC has a favorable prognosis [3]. Most cases present with a well-circumscribed, palpable breast mass with possible breast pain and nipple retraction [3].

Because there are currently no guidelines addressing male BACC (MBACC), diagnosis and treatment often rely on guidelines for female patients and case reports of male patients. We present a male with a nonhealing breast abscess who was ultimately diagnosed with BACC and treated with a modified radical mastectomy (MRM).

## Case report

**Figure 1:**
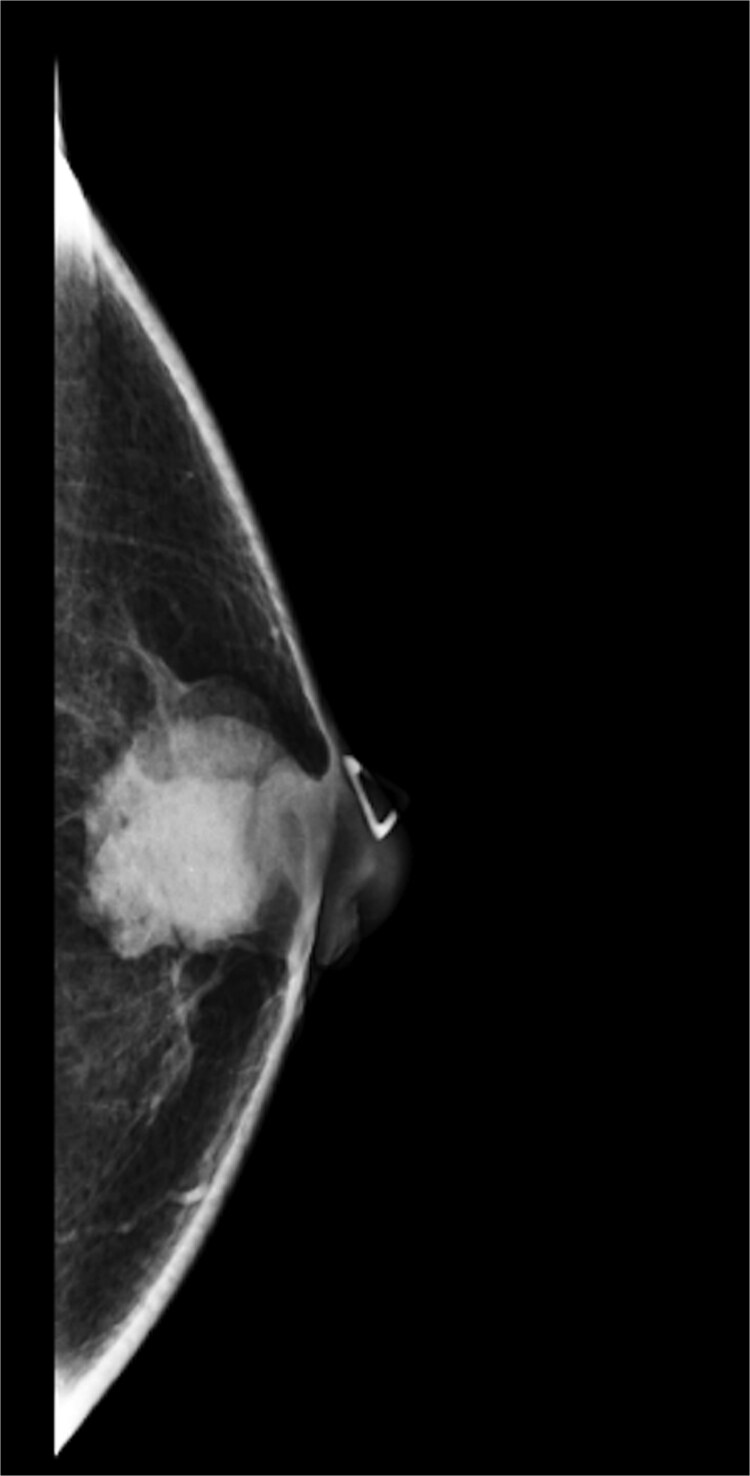
Diagnostic mammogram of left breast showing heterogenous retroareolar mass – first view.

**Figure 2:**
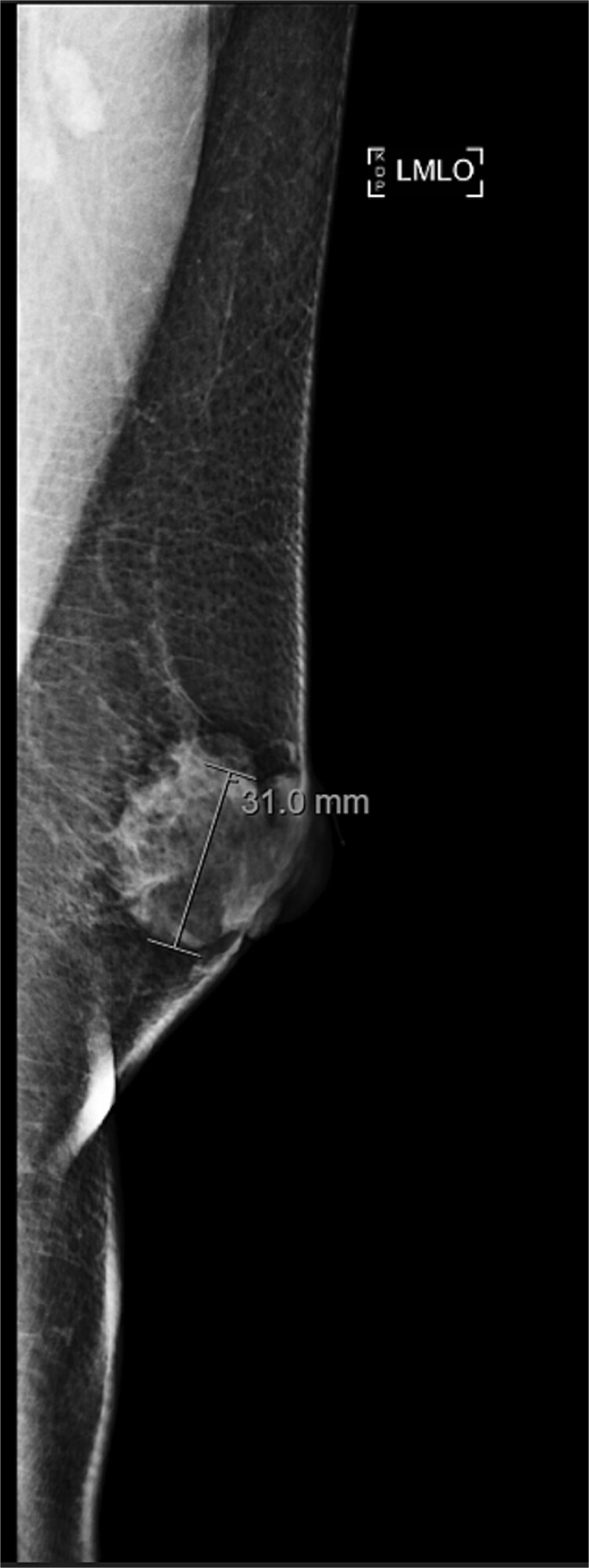
Diagnostic mammogram of left breast showing heterogenous retroareolar mass – second view.

A 63-year-old male with a past medical history of heavy daily tobacco use, COPD, and a left breast leiomyoma diagnosed in 2018 presented to a general surgeon with a nonhealing left breast abscess. The patient had a persistent 3 cm retroareolar mass despite multiple incision and drainage procedures. On exam, the left axillary lymph nodes were unremarkable and the nipple, while not inverted, was chronically distorted, indurated, and inflamed. The persisting mass and inflammation were initially concerning for inflammatory breast cancer. Superficial biopsy by both a dermatologist and general surgeon were equivocal. Diagnostic mammogram and targeted left breast ultrasound revealed a retroareolar 3.3 × 3.6 × 2.7 cm heterogenous mass, corresponding to the area of palpable concern (Figs 1 and 2).

**Figure 3:**
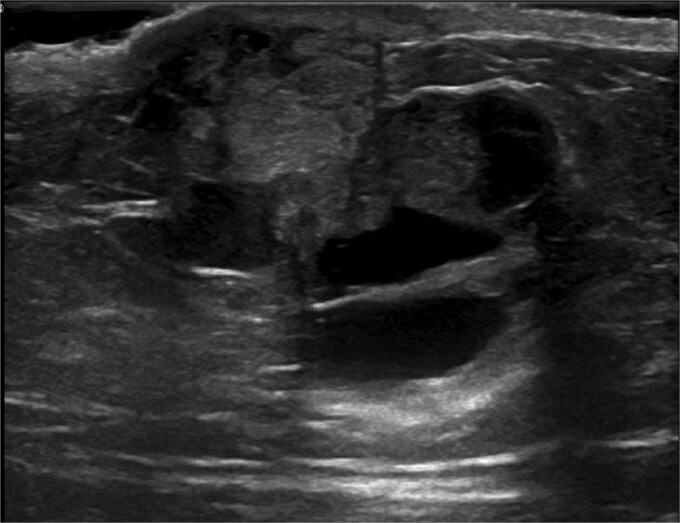
Ultrasound of retroareolar area of palpable concern – first view.

**Figure 4:**
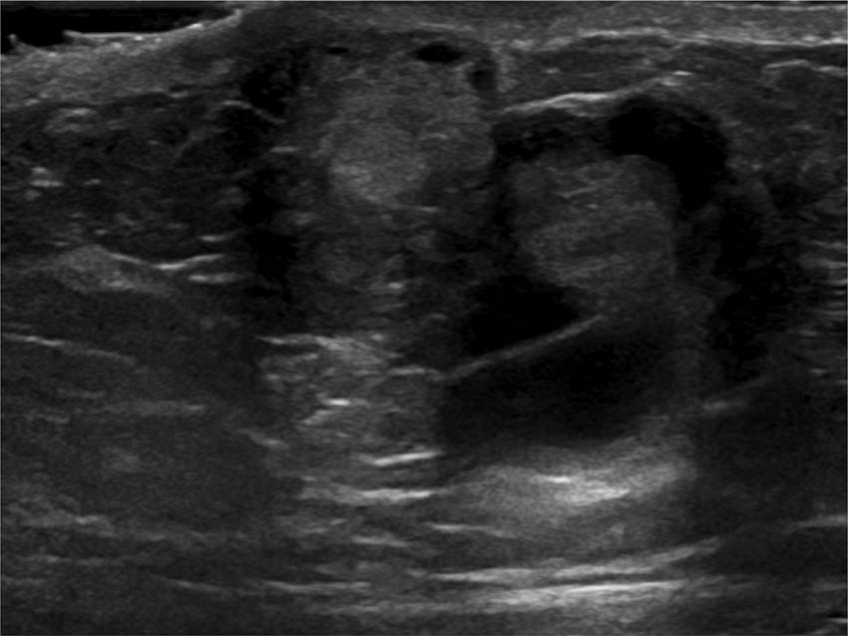
Ultrasound of retroareolar area of palpable concern – second view.

Ultrasound-guided core needle biopsy of the mass showed dissociated fragments of cribriform-like and papillary-like carcinoma (Figs 3 and 4).

The tumor was immunoreactive for CD117 (c-kit), CK5 (basal cytokeratin), and p40. Ki-67 expression was low at 38%. It was nonimmunoreactive for CD10 and the tumor was triple-receptor-negative. The pathologic diagnosis was unclear at this time, with the differential diagnosis including BACC and basal-like ductal carcinoma in situ (DCIS). Due to diagnostic uncertainty and the patient’s adamance against adjuvant therapy, MRM was performed. Surgical pathology revealed a 3.5 cm histologic grade 2 adenoid cystic carcinoma. All margins and all 13 lymph nodes were free of invasive carcinoma. The patient is currently without evidence of disease at four months after surgery.

## Discussion

We report the case of a 63-year-old man with histology-confirmed BACC, a rare occurrence. A retrospective study of 10% of the US population between 1997 and 2006 found only five men diagnosed with BACC [4]. A recent literature review details 18 published cases of MBACC up to 2022 [5]. There has been one other reported cause of MBACC [6].

ACC is a biphasic tumor, consisting of both luminal and myoepithelial (basaloid) cells [7]. BACC is histologically similar to ACC of the salivary glands and lungs [3]. Luminal cells line true glands, while myoepithelial cells line pseudocystic spaces [7]. Luminal cells express proteins such as E-cadherin, whereas myoepithelial-basaloid cells express proteins including Type IV collagen and fibronectin [7]. This preserved polarity and expression of proteins promoting cell differentiation may be the reason that BACC holds a favorable prognosis [7]. BACC’s growth pattern is usually cribriform, solid, and/or tubular [7]. Because of this, BACC can be misclassified as cribriform ductal carcinoma [3]. Our specimen had a mixed cribriform and papillary pattern, making the diagnosis difficult. Our specimen’s papillary pattern and cytokeratin positivity was one of the reasons basal-like DCIS remained on the differential [8].

Our patient represents the most common receptor phenotype seen with ACC—triple-receptor-negative [1, 7, 9]. However, hormone-receptor-positive BACC is not uncommon [4, 7, 9]. CK5 expression, which was present in our patient, as well as CK6, CK14, and CK17 expression are common in BACC [7].

A literature review including 18 cases of MBACC found that all patients presented with a breast mass, most of which were subareolar like our patient [5]. It is common for breast masses to be present for several years before BACC diagnosis [4]. Radiographic imaging is often nonspecific [3]. The median age at presentation among the men was 39, much younger than 60 in women [5]. Due to inadequate guidelines for MBACC, management relies on published cases. While local surgical therapy is the generally most common treatment for BACC [1], simple and modified radical mastectomies are preferred for MBACC [5]. In a review of MBACCs, both patients who underwent a lumpectomy experienced recurrence or distant metastasis [5]. This may be because the basaloid subtype of ACC tends to be infiltrative, making wide local excision inadequate. It is noteworthy that it is not specified if these patients received radiotherapy [5]. Although axillary staging for BACC is debated, we performed an axillary lymph node dissection since axillary node metastasis in both MBACC and basal-like BACC is not unheard of [10]. Three of 18 reported MBACC cases to date have metastasized to axillary lymph nodes [5]. The role of radiation and chemotherapy in MBACC remains unknown [5].

BACC has a favorable prognosis compared to salivary gland and lung ACC [11]. The 10-year survival rate of BACC is 90%–100%, with very low rates of nodal and distant metastases [7]. One study following 87 BACC patients found 18 recurrences, of which 14 were distant metastases [12]. Metastases are more common with basaloid BACC [12]. However, since data on MBACC is limited, caution should be taken when extrapolating data on survival and prognosis in female BACC to male patients. Five of the 19 reported cases of MBACC have either recurred locally or metastasized to distant sites, suggesting the possibility of worse outcomes in males [5, 6]. It is unclear whether this is due to increased aggressiveness of BACC in males or a lack of timely diagnosis.

In conclusion, we discussed the case of a 63-year-old man with BACC successfully treated with MRM. Due to its rarity, there is limited data regarding prognosis and effective treatment of MBACC. Based on the literature and our experience, MRM seems to be an effective treatment for MBACC [5]. While the importance of axillary surgery in BACC is unclear, there may be a role for it in male cases [5].

## References

[1] Macias J, Sales Nogueira Amorim Canedo F, Lu SE, et al. Treatment patterns of adenoid cystic carcinoma of the breast: a systematic review. J Clin Oncol 2024;42:e13147. 10.1200/JCO.2024.42.16_suppl.e13147.

[2] Dent R, Trudeau M, Pritchard KI, et al. Triple-negative breast cancer: clinical features and patterns of recurrence. Clin Cancer Res 2007;13:4429–34. 10.1158/1078-0432.CCR-06-3045. 17671126

[3] Boujelbene N, Khabir A, Boujelbene N, et al. Clinical review – breast adenoid cystic carcinoma. Breast 2012;21:124–7. 10.1016/j.breast.2011.11.006. 22154460

[4] Ghabach B, Anderson WF, Curtis RE, et al. Adenoid cystic carcinoma of the breast in the United States (1977 to 2006): a population-based cohort study. Breast Cancer Res 2010;12:R54. 10.1186/bcr2613 PMC2949643 20653964

[5] Wan D, Zhou H, Zhang Y. Adenoid cystic carcinoma of the breast in a male patient: a case report and literature review. Front Oncol 2022;12:905997. 10.3389/fonc.2022.905997. PMC9300960 35875113

[6] Mikhalchyshina IV, Kropelnytskyi VA, Deneka OO. Adenoid cystic carcinoma of the breast in a man. Ukrain J Clin Surg 2025;92:69–72. 10.26779/2786-832X.2025.1.69.

[7] Miyai K, Schwartz MR, Divatia MK, et al. Adenoid cystic carcinoma of breast: recent advances. World J Clin Cases 2014;2:732–41. 10.12998/wjcc.v2.i12.732. 25516849 PMC4266822

[8] Dabbs DJ, Chivukula M, Carter G, et al. Basal phenotype of ductal carcinoma in situ: recognition and immunohistologic profile. Mod Pathol 2006;19:1506–11. 10.1038/modpathol.3800678. 16941011

[9] Guldogan N, Esen G, Kayadibi Y, et al. Adenoid cystic carcinoma of the breast: multimodality imaging findings and review of the literature. Acad Radiol 2023;30:1107–17. 10.1016/j.acra.2022.10.003. 36357304

[10] Zhang M, Liu Y, Yang H, et al. Breast adenoid cystic carcinoma: a report of seven cases and literature review. BMC Surg 2022;22:113. 10.1186/s12893-022-01870-y. PMC8953026 35331206

[11] Li N, Xu L, Zhao H, et al. A comparison of the demographics, clinical features, and survival of patients with adenoid cystic carcinoma of major and minor salivary glands versus less common sites within the surveillance, epidemiology, and end results registry. Cancer 2012;118:3945–53. 10.1002/cncr.26740. 22179977 PMC3412946

[12] Slodkowska E, Xu B, Kos Z, et al. Predictors of outcome in mammary adenoid cystic carcinoma: a multi-institutional study. Am J Surg Pathol 2022;44:214–23. 10.1097/PAS.0000000000001378 31567278

